# Supplementary material for: Industrial Silicon-Wafer-Wastage-Derived Carbon-Enfolded Si/Si-C/C Nanocomposite Anode Material through Plasma-Assisted Discharge Process for Rechargeable Li-Ion Storage
Source: Nanomaterials (Basel). 2022 Feb 16;12(4):659. doi: 10.3390/nano12040659 (PMC8878063; doi:10.3390/nano12040659)
Supplement: Supplementary file 1 [file nanomaterials-12-00659-s001.zip › nanomaterials-1595974-supplementary.pdf]

Supplementary Materials

Industrial Silicon-Wafer-Wastage-Derived Carbon-Enfolded Si/Si-C/C Nanocomposite Anode Material through Plasma-Assisted Discharge Process for Rechargeable Li-Ion Storage

Rasu Muruganantham [1], Chih-Wei Yang [2], Hong-Jyun Wang [2], Chia-Hung Huang [3,4] and Wei-Ren Liu [1,*]

1 Department of Chemical Engineering, R&D Center for Membrane Technology, Center for Circular Economy, Chung Yuan Christian University, 200 Chung Pei Road, Chung Li District, Taoyuan City 32023, Taiwan; rmurugaphd@gmail.com
2 Sino Applied Technology Co., Ltd., Chung Li District, Taoyuan City 320030, Taiwan; tyang89@gmail.com (C.-W.Y.); hongjyunwa@gmail.com (H.-J.W.)
3 Department of Electrical Engineering, National University of Tainan, No.33, Sec. 2, Shulin St., West Central District, Tainan City 700, Taiwan; chiahung@mail.mirdc.org.tw
4 Metal Industries Research and Development Centre, Kaohsiung 70101, Taiwan
* Correspondence: wrliu1203@gmail.com; Tel.: +886-3-2653315; Fax: 886-3-2653399

1. Experimental physical and chemical characterization conditions

The crystal phase structure and purity were determined through X-ray powder diffraction (XRD) by using a Bruker D8 diffractometer with monochromatic CuK$_\alpha$ radiation. The diffractometer was operated at 40 kV and 20 mA with a wavelength (λ) of 1.54060 Å. Diffractiondata were recorded in a range 2θ = 10°–80°. The morphology of powders was investigated using a high-resolution transmission electron microscopic (HR-TEM, JEM2100). A Tristar 3000 was employed to accelerate the surface area, and a porosity instrument was used to measure the N_2 adsorption/desorption isotherms of the prepared product.

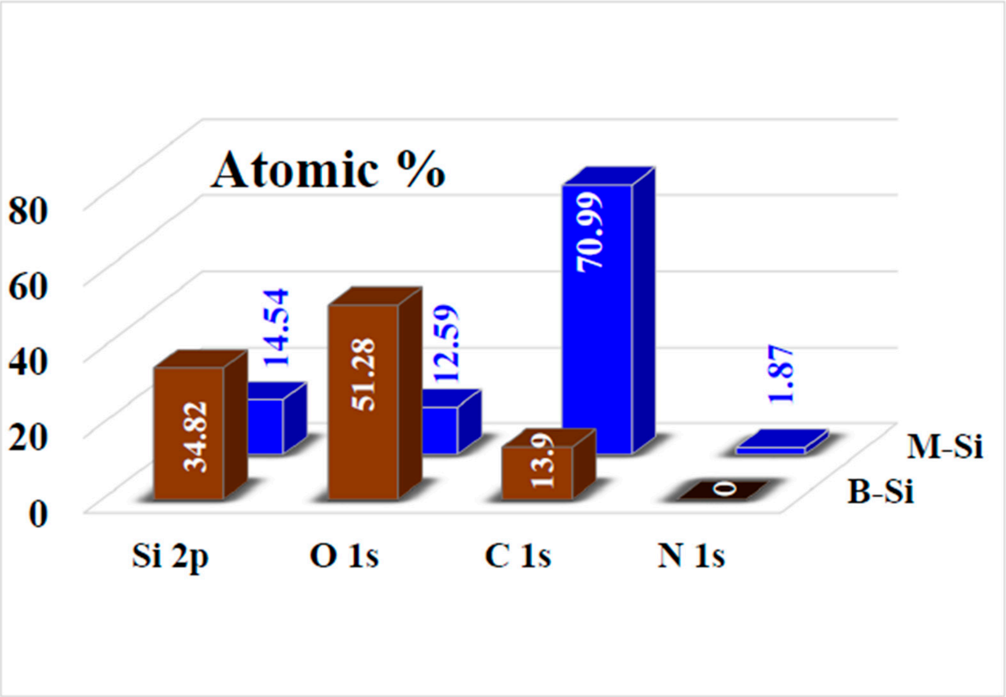

Figure S1. The M-Si and B-Si samples presence of elements atomic percentage estimated from XPS survey scan.

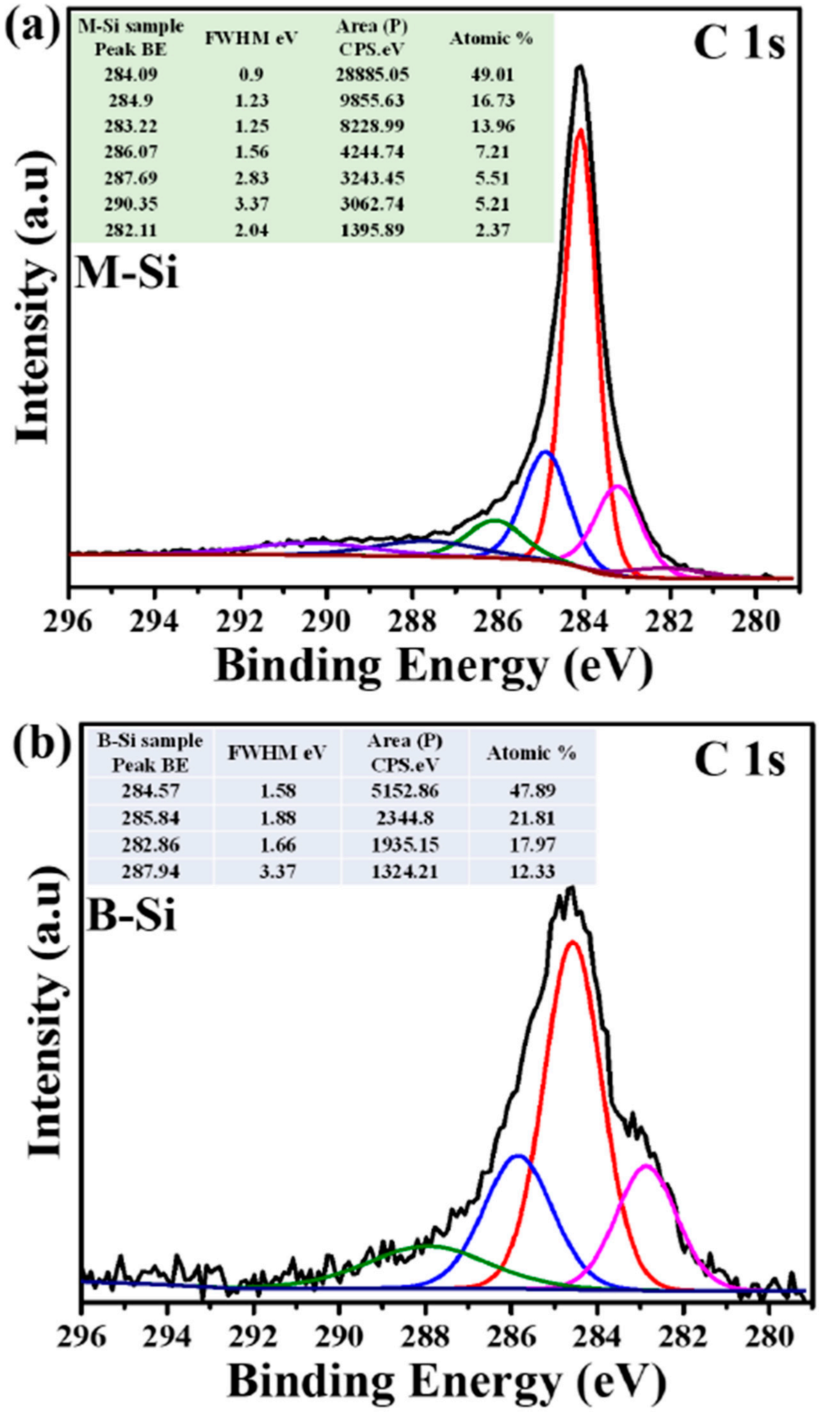

Figure S2. High-resolution C 1s XPS with deconvolved fitted spectra of **(a)** M-Si and **(b)** B-Si samples, respectively.

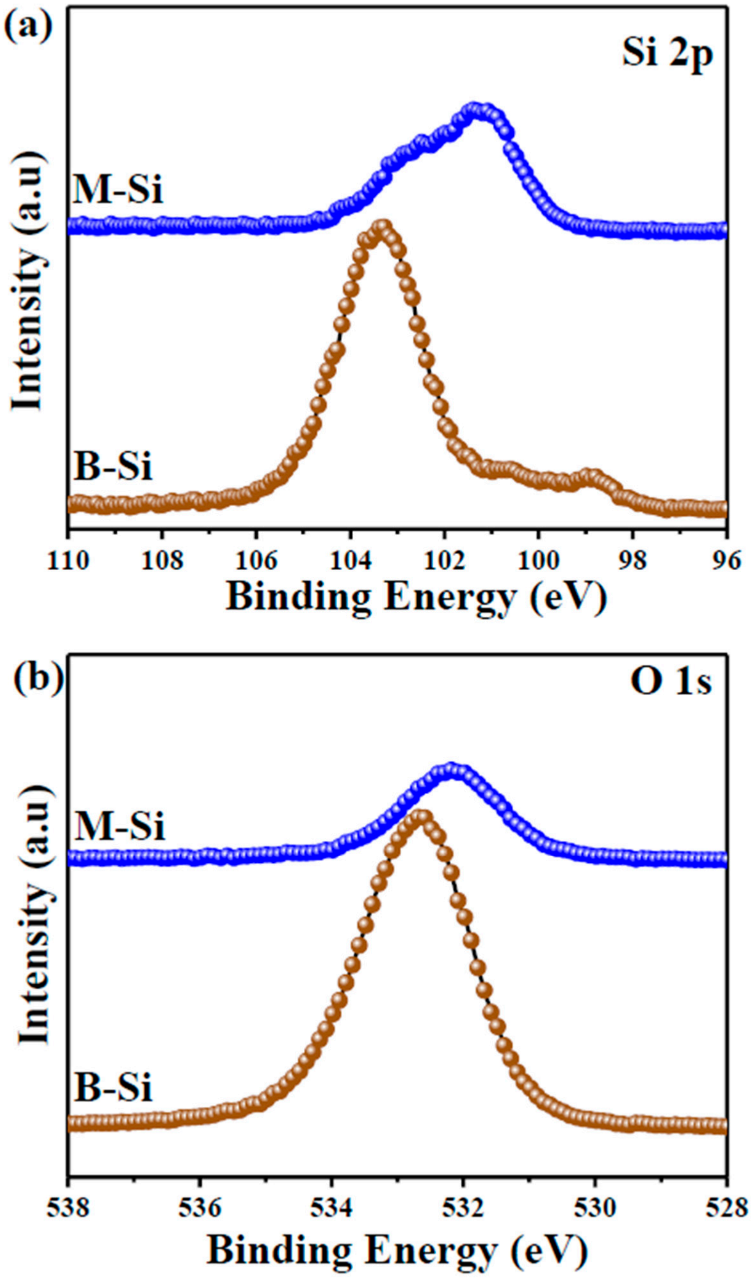

Figure S3. High-resolution O 1s XPS of **(a)** M-Si and **(b)** B-Si samples.

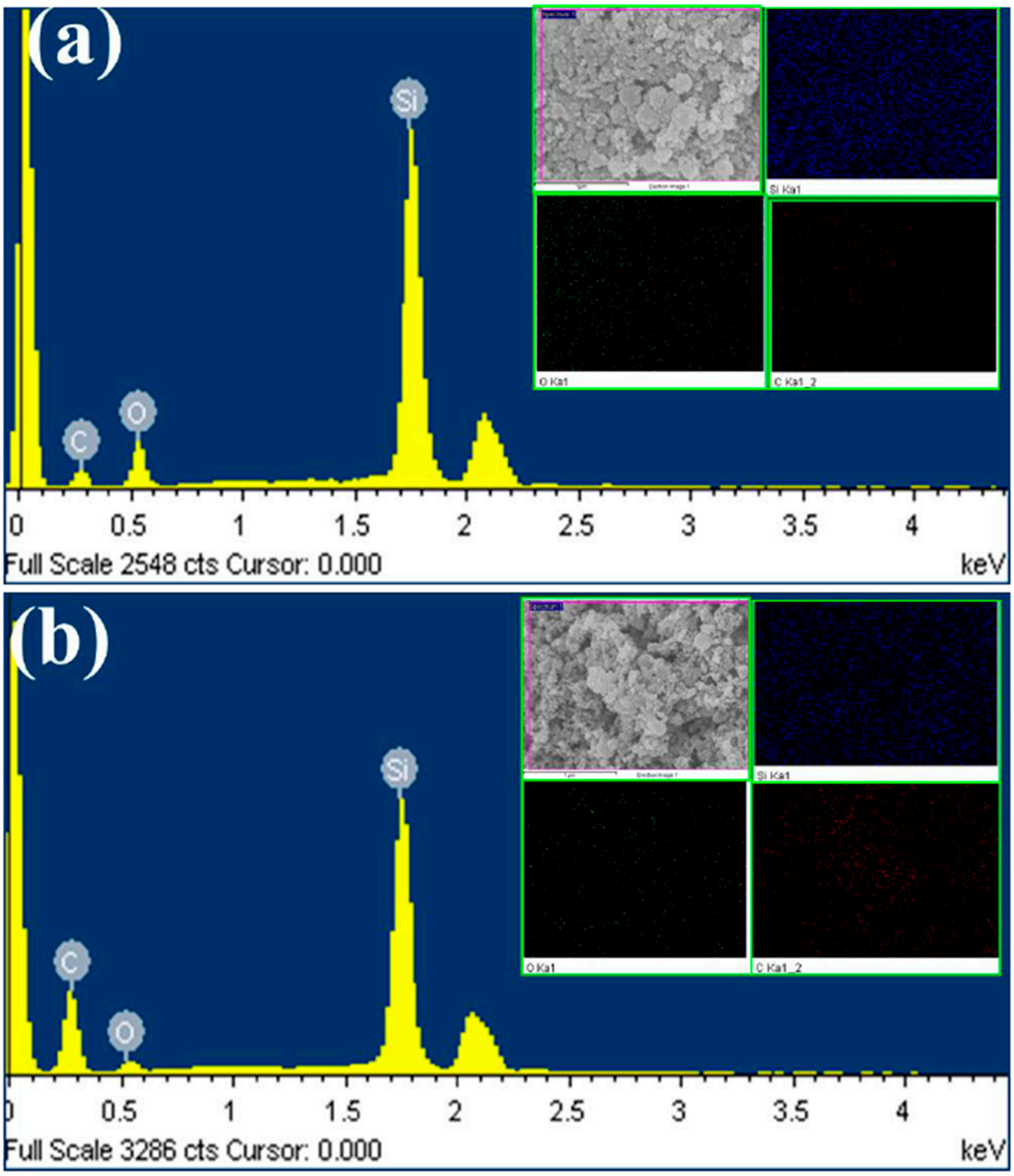

Figure S4. FE-SEM coupled EDX spectra of **(a)** M-Si and **(b)** B-Si samples, inset the corresponding E-mapping images.
